# Assessment of Safety and Probiotic Traits of *Enterococcus durans* OSY-EGY, Isolated From Egyptian Artisanal Cheese, Using Comparative Genomics and Phenotypic Analyses

**DOI:** 10.3389/fmicb.2020.608314

**Published:** 2020-12-11

**Authors:** Walaa E. Hussein, Ahmed G. Abdelhamid, Diana Rocha-Mendoza, Israel García-Cano, Ahmed E. Yousef

**Affiliations:** ^1^Department of Food Science and Technology, The Ohio State University, Columbus, OH, United States; ^2^Department of Microbiology and Immunology, National Research Center, Giza, Egypt; ^3^Botany and Microbiology Department, Faculty of Science, Benha University, Benha, Egypt; ^4^Department of Microbiology, The Ohio State University, Columbus, OH, United States

**Keywords:** probiotics, *Enterococcus durans*, genome mining, safety, bioinformatics

## Abstract

An *Enterococcus durans* strain, designated OSY-EGY, was previously isolated from artisanal cheese. In this work, comparative genomic and phenotypic analyses were utilized to assess the safety characteristics and probiotic traits of the bacterium. The comparative genomic analysis revealed that the strain is distantly related to potentially pathogenic *Enterococcus* spp. The genome was devoid of genes encoding acquired antibiotic resistance or marker virulence factors associated with *Enterococcus* spp. Phenotypically, the bacterium is susceptible to vancomycin, ampicillin, tetracycline, chloramphenicol, and aminoglycosides and does not have any hemolytic or gelatinase activity, or cytotoxic effect on Caco-2 cells. Altogether, these findings confirm the lack of hazardous traits in *E. durans* OSY-EGY. Mining *E. durans* OSY-EGY genome, for probiotic-related sequences, revealed genes associated with acid and bile salts tolerance, adhesion, competitiveness, antioxidant activitiy, antimicrobial activity, essential amino acids production, and vitamins biosynthesis. Phenotypically, *E. durans* OSY-EGY was tolerant to acidic pH (3.0), and presence of 0.3% bile salts. The bacterium showed adhesion capability to Caco-2 cells, cholesterol-lowering effect, DPPH scavenging activity, and antimicrobial activity against several Gram-positive pathogenic bacteria. Based on the current work, we propose that *E. durans* OSY-EGY is a potentially safe strain with desirable probiotic and antimicrobial traits. Thus, the investigated strain could be a promising candidate for several industrial applications.

## Introduction

Enterococci are non-spore forming bacteria with Gram-positive cell wall and fermentative metabolism (Ludwig et al., 2009). They are characterized by their tolerance to heat, salinity and extreme pH (Hanchi et al., 2018). Enterococci could be found in fermented food products such as cheeses, sausages, olives and vegetables (Franz et al., 1999; Giraffa, 2002). The occurrence of enterococci in food has been viewed for long time as an indicator of poor manufacturing practice and product contamination (Thompson and Marth, 1986; Halkman and Halkman, 2014); however, other researchers consider enterococci to have a minimum role as a hygienic index in the food industry (Birollo et al., 2001). Presence of enterococci in raw-milk-cheeses at populations up to 10^8^ CFU/g suggests their commensal nature or their role as natural starter culture (Giraffa et al., 1997). These bacteria may have a role in ripening, aroma development and shelf-life improvement of fermented foods (Foulquié Moreno et al., 2006). Some strains produce enterocins, which are antimicrobial peptides active against some foodborne pathogens and spoilage bacteria (Khan et al., 2010).

Desirable probiotic characteristics have been reported in some enterococcal strains; these include their ability to inhabit human and animals gastrointestinal tract (GIT) and effectively participate in developing gut microbiome (Dominguez-Bello et al., 2010). Enterococcal strains also may exert their probiotic functions throughout immune system regulation, antioxidant activity, maintaining normal intestinal microflora, and lowering cholesterol levels (Franz et al., 2011). When ingested in high numbers, some enterococci may improve remission of irritable bowel syndrome or antibiotic-associated diarrhea (Gaggìa et al., 2010; Kreuzer et al., 2012). *Enterococcus faecalis* and *E. faecium* were used as commercial probiotics or feed additives (Franz et al., 2011) whereas other species such as *E. durans* and *E. hirae* were proposed for such use (Nami et al., 2014, 2019; Pieniz et al., 2014). Examples of commercial enterococci probiotic preparations are *E. faecium* NCIMB 10415(SF68), used in Cernivet^®^ (Cerbios-Pharma SA, Switzerland) and Cylactin^®^ (Hoffmann-La Roche, Basel, Switzerland), and *E. faecalis* Symbioflor^®^ 1 used in Symbioflor 1 (Symbiopharm, Herborn, Germany) (Serio et al., 2010; Baccouri et al., 2019).

Despite these beneficial uses, some enterococcal strains have been associated with human diseases and nosocomial infections. These strains can cause bacteremia, urinary tract infection, endocarditis, surgical site infection, and diarrhea (Murray and Weinstock, 1999; Kayser, 2003; Klare et al., 2003; Werner et al., 2011). Potentially pathogenic enterococci have been associated with hospital infections, but these are rarely associated with infections outside healthcare settings (Sanders et al., 2010). It has been proposed that the use of broad spectrum antibiotics, catheters, immune-suppressors or chemotherapy affects host-microbe balance and facilitates infection with some enterococci, mostly *E. faecalis* and *E. faecium* (Murray, 2000; Ubeda et al., 2010). Potentially pathogenic enterococci usually carry clinically relevant antibiotic resistances and marker virulence factors (Ike, 2017). Considering these findings, *Enterococcus* spp. are neither recommended for the “qualified presumption of safety” list of biological agents by the European Food Safety Authority (EFSA) nor have the generally recognized as safe (GRAS) status from the US Food and Drug Administration (Hanchi et al., 2018). For distinction between commensal/beneficial and potentially pathogenic enterococcal strains, comprehensive assessment of their safety characteristics is necessary (EFSA, 2012a,b).

The rise of antimicrobial resistance in foodborne pathogens and the need for natural food bio-preservatives prompted the search for new strains capable of producing potent antimicrobial agents. *E. durans* OSY-EGY, used in this study was isolated from artisanal cheese and showed a promising antimicrobial activity against selected pathogenic species (Hussein et al., 2019). The current study was initiated to thoroughly explore the safety, antimicrobial and probiotic traits of *E. durans* OSY-EGY and its potential usefulness to the industry.

## Materials and Methods

### Bacterial Strains and Culture Conditions

*E. durans* OSY-EGY was streaked on de Man, Rogosa and Sharpe (MRS) agar (Oxoid, Thermo scientific, Waltham, MA, United States), and its overnight subculture in MRS broth was mixed with 80% sterile glycerol in a 1:1 ratio and stocked at −80°C. In preparation for experiments, frozen stock culture was streaked on MRS agar and inoculated plates were incubated at 30°C for 48 h followed by cultivation in MRS broth at 30°C for 18 h. *E. faecalis* Symbioflor 1 strain was isolated from Symbioflor^®^ 1 commercial preparation (SymbioPharm, Herborn, Germany), by streaking on MRS agar and incubation at 30°C for 48 h followed by cultivation in MRS broth at 30°C for 18 h. The control strains, *Bacillus cereus* ATCC 14579, *Escherichia coli* K12, *Staphylococcus aureus* ATCC 25923, *E. faecalis* ATCC 29212 and *E. coli* BL21-pET22 (+) (Invitrogen, Carlsbad, CA, United States), *Listeria monocytogenes* Scott A, and *S. aureus* ATCC 29213 were obtained from the culture collection of the food microbiology laboratory, at the Ohio State University (Columbus, OH, United States).

### Genotypic Characterization

#### Screening *E. durans* OSY-EGY Genome for Safety- and Probiotic-Related Traits

For safety assessment, *E. durans* OSY-EGY genome (Hussein et al., 2019) was screened using the Comprehensive Antibiotic Resistance Database (CARD) (Jia et al., 2017) and Virulence Factor of Bacterial Pathogens Database (VFDB)^1^ (Chen et al., 2005). Additionally, *E. durans* OSY-EGY genome was mined for genes associated with biosynthetic pathways of biogenic amines using KAAS-KEGG Automatic Annotation Server^2^ (Moriya et al., 2007) and KEGG Mapper^3^ (Kanehisa et al., 2019). The genome was also screened for the presence of Clustered Regularly Interspersed Short Palindromic Repeats (CRISPR) using CRISPR Finder^4^ (Grissa et al., 2007). For beneficial traits, *E. durans* OSY-EGY draft genome was mined for genes associated with biosynthetic pathways of essential amino acids and vitamins using KAAS-KEGG Automatic Annotation Server (see text footnote 2) (Moriya et al., 2007) and KEGG Mapper (see text footnote 3) (Kanehisa et al., 2019).

#### Comparative Genomic Analysis

For comparative genomic analysis, the genomic sequences of eleven strains were selected to represent either probiotic or potentially pathogenic enterococci. Group 1, representing probiotic enterococci, included four commercial probiotic strains (*E. faecium* T110, *E. faecium* L3, *E. faecium* LX, and *E. faecalis* Symbioflor 1) and two potential probiotic strains (*E. durans* KLDS6.0930 and *E. faecium* 17OM39). Group 2, representing potentially pathogenic enterococci, included five strains (*E. faecium* DO, *E. faecium* 6E6, *E. faecium* Aus0085, *E. faecium* Aus0004, and *E. faecium* E39). The chromosomal genome sequences of these eleven strains were downloaded from the GenBank database using their GenBank accession numbers listed in Table 1. *E. durans* OSY-EGY draft genome was compared with these selected genomes based on the presence of antibiotic resistance, virulence genes, genes associated with acid tolerance (Ladero et al., 2013; Liu et al., 2016), bile salts tolerance (Budin-Verneuil et al., 2007; Hamon et al., 2011), cellular adhesion (Ladero et al., 2013; Hymes and Klaenhammer, 2016; Liu et al., 2016), competitiveness and persistence (Ghattargi et al., 2018), and antioxidant effects (Li et al., 2018a), retrieved from literature and analyzed using online NCBI BLAST tools; BLASTN for nucleotide sequences (Chen et al., 2013) or TBLASTN for amino acid sequences (Gertz et al., 2006). The presence and absence of genes, in the compared genomes, were visualized as heatmaps constructed using R^5^.

**TABLE 1:** Genomes of *Enterococcus durans* OSY-EGY and probiotic and potentially pathogenic members of the genus.

**NCBI^*a*^ strain identifiers**	**Source**	**GenBank accession**	**Genome size (Mb)**	**GC%**	**CDS**	**References**
*E. durans* OSY-EGY	Artisanal cheese	GCA_004330425.1	3.2	37.69	3487	Hussein et al., 2019
**Potential probiotic**						
*E. durans* KLDS6.0930	Fermented cream	CP012384.1	2.8	38.0	2569	Li et al., 2018a
*E. faecium* 17OM39	Feces	GCA_001652715.1	2.8	38.5	2639	Ghattargi et al., 2018
**Commercial probiotic**						
*E. faecium* T110	Commercial preparation	CP006030.1	2.7	38.4	2522	Natarajan and Parani, 2015
*E. faecium* L3	Commercial preparation	GCA_000787055.1	2.6	38.0	2559	Suvorov et al., 2019
*E. faecium* LX	Commercial preparation	GCA_000787065.1	2.7	38.3	2641	Suvorov et al., 2019
*E. faecalis* Symbioflor1	Commercial preparation	GCA_000317915.1	2.8	37.7	2810	Domann et al., 2007
**Potentially pathogenic**						
*E. faecium* DO	Blood	CP003583.1	2.6	37.9	2703	Lam et al., 2012
*E. faecium* 6E6	Feces	CP013994.1	2.9	37.6	3307	Geldart and Kaznessis, 2017
*E. faecium* Aus0085	Blood	CP006620.1	2.9	37.9	2938	Zhong et al., 2017
*E. faecium* Aus0004	Blood	CP003351.1	2.9	38.3	2860	Zhong et al., 2017
*E. faecium* E39	Blood	CP011281.1	2.7	37.8	2907	Geldart and Kaznessis, 2017

*^*a*^National Center for Biotechnology Information.*

To decipher the genomic difference between *E. durans* OSY-EGY and five genomes of the potentially pathogenic group, BLAST atlas was generated to align the query genomes using *E. faecium* DO as a reference. The BLAST atlas was drawn using BLAST Ring Image Generator (BRIG) (Alikhan et al., 2011).

Genomes of *E. durans* OSY-EGY and six potential or commercial probiotic enterococci were additionally compared. BLAST atlas was generated to align the query genomes using *E. durans* OSY-EGY as a reference. Sequences encoding bacteriocins were searched in the query genomes using the antibiotics and secondary metabolite analysis shell, antiSMASH v4.1.0 (Blin et al., 2018) and the web-based bacteriocin genome mining tool, BAGEL v4.0 (Walsh et al., 2011).

#### Detection of Selected Virulence Genes by Polymerase Chain Reaction (PCR)

PCR analysis was conducted on *E. durans* OSY-EGY and *E. faecalis* Symbioflor 1 to assess the presence of selected enterococcal virulence determinants and genes encoding biogenic amines. The analysis covered the following groups of genes; (i) genes associated with tissue damage that include *cylA* (cytolysin), *gelE* (gelatinase), and *hyl* (hyaluronidase); (ii) genes involved in adhesion and colonization, which include *asa1* and *agg* (aggregation substances), *esp* (enterococcal surface protein), *ace* (collagen adhesion protein), and *efaA* (endocarditis antigen); and (iii) genes encoding biogenic amines, i.e., *hdc* (histidine decarboxylase), *tdc* (tyrosine decarboxylase) and *odc* (ornithine decarboxylase). For Genomic DNA (gDNA) extraction, *E. durans* OSY-EGY and *E. faecalis* Symbioflor 1 were grown in MRS broth at 37°C for 16 h. Aliquots (1 ml) of each culture were centrifuged at 10,000 × *g* for 10 min at 4°C (Model 5415R: Eppendorf, Hauppauge, NY, United States) and cell pellets were washed two times with sterile saline solution. The gDNA was isolated from the cell pellets using genomic DNA purification kit (Wizard^®^ kit; Promega, Madison, WI, United States) following the manufacturer’s protocol. PCR amplification was performed in 30 μL reaction mixture containing 1 μg of gDNA, 1 × PCR buffer, 1.5 mM MgCl_2_, 0.2 mM of dNTP mix, forward primer, and reverse primer and 2 U Taq DNA polymerase (Platinum^®^; Thermo Fisher Scientific, Waltham, MA, United States). Genes, corresponding primers, primer concentration, product size and the PCR conditions applied are presented in Table 2. Amplifications were done by a thermal cycler (CFX96 Real-Time System; Bio-Rad, Hercules, CA, United States). Amplicon visualization was done with DNA gel stain (SYBER safe; Invitrogen) under UV light using gel documentation system (ChemiDoc; Bio-Rad).

**TABLE 2:** Use of PCR in the detection of virulence and biogenic amines production genes in *Enterococcus durans* OSY-EGY and *E. faecalis* Symbioflor 1.

**Gene**	**Protein**	**Primers**	**Primer concentration (μM)**	**PCR conditions**	**Product size (bp)**
*cylA*	Cytolysin	F: ACT CGG GGA TTG ATA GGC R: GCT GCT AAA GCT GCG CTT	0.28	(94°C 1 min, 56°C 1 min, 72°C 2 min) *30	688
*gelE*	Gelatinase	F: TATGACAATGCTTTTTGGGAT R: AGATGCACCCGAAATAATATA	0.1		213
*hyl*	Hyaluronidase	F: ACAGAAGAGCTGCAGGAAATG R: GACTGACGTCCAAGTTTCCAA	0.14		276
*asa1*	Aggregation Substances	F: GCACGCTATTACGAACTATGA 375 R: TAAGAAAGAACATCACCACGA	0.14		375
*esp*	Enterococcal Surface Protein	F: AGATTTCATCTTTGATTCTTGG R: AATTGATTCTTTAGCATCTGG	0.28		510
*ace*	Collagen Adhesin Protein	F: GAA TTG AGC AAA AGTT CAA TCG R: GTC TGT CTT TTC ACT TGT TTC	0.1	(94°C 1 min, 55°C 1 min, 72°C 2 min) *30	1008
*efaA*	Endocarditis Antigen	F: GCCAATTGGGACAGACCCTC R: CGCCTTCTGTTCCTTCTTTGGC	0.1		688
*Agg*	Aggregation Substances	F: AAG AAA AAG AAG TAG ACC AAC R: AAA CGG CAA GAC AAG TAA ATA	0.4	(94°C 1 min, 52°C 1 min, 72°C 2 min) *30)	1553
*hdc*	Histidine Decarboxylase	F: AGATGGTATTGTTTCTTATG R: AGACCATACACCATAACCTT	0.3		367
*tdc*	Tyrosine Decarboxylase	F: GAYATNATNGGNATNGGNYTNGAYCARG R: CCRTARTCNGGNATAGCRAARTCNGTRTG	1		924
*odc*	Ornithine decarboxylase	F: GTNTTYAAYGCNGAYAARACNTAYTTYGT R: ATNGARTTNAGTTCRCAYTTYTCNGG	2		1446

#### Plasmid Isolation

*E. durans* OSY-EGY was screened for presence of plasmids. *E. faecalis* Symbioflor 1 was used as negative control (Domann et al., 2007) and *Escherichia coli* BL21-pET22(+) was used as positive control. *E. durans* OSY-EGY and *E. faecalis* Symbioflor 1 were grown in MRS broth at 37°C for 16 h. *E. coli* was grown in Luria-Bertani broth (Sigma-Aldrich, St. Louis, MO, United States) at 37°C with orbital shaking at 200 rpm for 16 h. Aliquots (1 ml) of each culture were centrifuged at 8,000 × *g*, for 10 min (Model 55415R; Eppendorf), the supernatants were discarded, and cell pellets were used for the subsequent plasmid isolation. Plasmid DNA extraction was performed using a plasmid extraction kit (PureYield^TM^ Plasmid Miniprep System; Promega), following the instructions of the manufacturer. DNA gel stain (SYBR Safe; Invitrogen) was used for plasmid visualization under UV light (ChemiDoc; Bio-Rad) for image analysis.

### Phenotypic Safety Assessment

#### Antibiotic Susceptibility

The minimum inhibitory concentration (MIC) of 9 antibiotics (ampicillin, kanamycin, vancomycin, clindamycin, tetracycline, erythromycin, gentamycin, streptomycin, and chloramphenicol) was determined against *E. durans* OSY-EGY strain using the broth microdilution method (Wikler et al., 2006). Briefly, two-fold serially diluted antibiotics, at final concentrations of 1, 2, 4, 8, 16, 32, 64, 128, 256, 512, and 1,024 μg/ml, were prepared before 50-μl aliquots of the antibiotics’ preparations were added into 96-well plates (Corning, Tewksbury, MA, United States). This was followed by adding equal volume of *E. durans* OSY-EGY culture suspension that was adjusted to OD_600_ of 0.001 in Muller Hinton broth (BD Diagnostic, Franklin Lakes, NJ, United States). The MIC of each antibiotic was determined as the lowest concentration at which no growth of the OSY-EGY strain was observed following incubation for 24 h at 37°C. Interpretations for susceptibility to each antibiotic was based on the guidelines adopted by EFSA (2012a). The test was performed in triplicate.

#### Gelatinase Activity

*E. durans* OSY-EGY was tested for gelatinase production using a method described previously (Su et al., 1991) with some modifications. Briefly, single colony of *E. durans* OSY-EGY was streaked on nutrient agar (BD Diagnostic) containing 3% gelatin (BD Diagnostic) and incubated at 37°C for 24 h. After incubation, saturated solution of ammonium sulfate (Fisher scientific, Pittsburgh, PA, United States) was poured on the surface of the medium to precipitate the non-hydrolyzed gelatin. Transparent haloes around the colonies were indicative of the gelatinase activity. *B. cereus* ATCC 14579 was used as a positive control and *E. coli* K12 was used as a negative control. The test was repeated three times and averages were reported.

#### Hemolysin Activity

Hemolysin activity (Eaton and Gasson, 2001) was tested after growing culture of *E. durans* OSY-EGY overnight at 37°C on tryptic soy agar supplemented with 5% sheep blood (Remel San Diego, CA, United States). *S. aureus* ATCC 25923 was used as positive control for β hemolysis and *E. faecalis* ATCC 29212 was used as negative control. The test was repeated three times and averages were reported.

#### Determination of Cytotoxicity

Human colorectal adenocarcinoma cell line Caco-2 cells (HTB-37^TM^, American Type Culture Collection, Manassas, VA, United States) were cultured in Dulbecco’s modified Eagle’s medium (DMEM) (Thermo Fisher Scientific) containing 4.5 g/L glucose and supplemented with 15% heat-inactivated fetal bovine serum (FBS), 1% non-essential amino acids, 1% L-glutamine, and 1% penicillin-streptomycin (Gibco^TM^, Thermo Fisher Scientific). The cells were incubated at 37°C with 5% CO_2_. Caco-2 cells were grown in T-75 flasks (Corning^®^, Corning, NY, United States) for 4–6 days until reaching 70–90% confluence, then dispersed using 0.05% trypsin-0.5 mM EDTA (Gibco, Thermo Fisher Scientific). Cells were sub-cultured in the appropriate plates depending on the experiment to be performed. After reaching 100% confluency, the cells were incubated at 37°C for 11–14 days, a period at which they become highly differentiated. The medium was refreshed every second day with fresh DMEM medium. At 100% confluency, the content of the FBS was decreased to 7.5% (v/v).

Caco-2 cells were seeded in 96-well culture plate (Corning^®^), at a density of 2 × 10^3^ cells/well and incubated as described earlier until post-confluence. DMEM medium was removed and Caco-2 cells were washed with calcium and magnesium-free Dulbecco’s phosphate buffered saline (DPBS) (Gibco, Thermo Fisher Scientific) followed by adding DMEM containing 1% (v/v) FBS.

*E. durans* OSY-EGY and *E. faecalis* Symbioflor 1 were cultured in MRS broth at 30°C for 18 h, the cultures were centrifuged at 16,000 × *g* for 2 min and the resulting cell pellets were washed two times in DPBS. Antibiotic-FBS-free DMEM was used to resuspend and dilute the cell pellets to final cell density of 10^8^ CFU/ml. Caco-2 cells were then exposed to *E. durans* OSY-EGY or *E. faecalis* Symbioflor 1 cells and the plate was incubated at 37°C for 24 h. After incubation, the cytotoxicity was evaluated by measuring the activity of lactate dehydrogenase (LDH) released in the culture media by the damaged cells using cytotoxicity detection kit (Roche, Basel, Switzerland) following manufacturer’s recommendations. The final absorbance was measured at 490 nm and 680 nm (test and reference wavelengths, respectively) using a microplate reader (Multiskan GO, Thermo Scientific). The results were expressed as percent of the positive control (Caco-2 cells exposed to 1% of Triton X-100). The Caco-2 cells was also observed using inverted microscope after 3 h of exposure to *E. durans* OSY-EGY or *E. faecalis* Symbioflor 1 cells suspended in DMEM to final cell density of 10^6^ CFU/ml, compared to untreated Caco-2 cells.

### Phenotypic Assessment of Probiotic Characteristics

#### Acid Tolerance

The acid tolerance of *E. durans* OSY-EGY was tested as previously described (Ramos et al., 2013) with some modifications. *E. durans* OSY-EGY was cultured in MRS broth at 30°C for 18 h followed by centrifugation at 16,000 × *g* for 10 min at 4°C (Model 55415R; Eppendorf). The cell pellets were resuspended in a freshly pH-adjusted MRS. The MRS was adjusted to pH 2.0 and pH 3.0 using 1 N HCl (Fisher Scientific) to mimic the gastric acidic conditions and to pH 7.0 using 1N NaOH (Fisher Scientific) as control. The final cell density was adjusted to approximately 10^8^ CFU/ml. After incubation at 37°C for 2 h, 1-ml samples were taken immediately before and after the incubation, and tenfold serially diluted before spread-plating on MRS agar. After incubation at 30°C for 48 h, the colonies were counted and expressed as Log_10_ CFU/ml. The assay was performed in duplicate and repeated three times.

#### Bile Salts Tolerance

For testing the bile tolerance, the previously described method (Walker and Gilliland, 1993) was followed with some modifications. Fresh 18 h culture of *E. durans* OSY-EGY was inoculated, at final cell density of 10^8^ CFU/ml, in MRS broth supplemented with 0.3% bile salts (Oxgall, Sigma-Aldrich). Cells grown in MRS broth without bile salt was used as control. Culture aliquots, 200 μl each, were distributed into 96-well microplate (Corning) before incubation at 37°C for 9 h in anaerobic jar to simulate human intestinal conditions. Bacterial growth was monitored by measuring the culture’s optical density at 600 nm (OD_600_) hourly using microplate reader (UV max; Molecular Devices). The measured absorbance values were plotted against the time of incubation and compared with the control. The assays were performed in triplicate and repeated three times.

#### Bacterial Adhesion to Caco-2 Cells

Caco-2 cells were seeded in 12-well culture plate (Corning^®^), at a density of 1 × 10^5^ cells/well, and incubated as described earlier until post-confluence. DMEM medium was removed, and the monolayer of Caco-2 cells were washed two times with antibiotic-FBS-free DMEM. *E. durans* OSY-EGY or *E. faecalis* Symbioflor 1 were cultured in MRS broth at 30°C for 18 h, cultures were centrifuged at 16,000 × *g* for 2 min and the cell pellets were washed two times in DPBS. Antibiotic-FBS-free DMEM was used to resuspend and dilute the cell pellets to a final cell density of 10^6^ CFU/ml. The Caco-2 cell were co-cultured with 1 mL of bacterial cell suspension and incubated 37°C for 3 h. The medium was aspirated to remove the non-adhering bacterial cells, and Caco-2 cells were rinsed three times with 1 mL of DPBS. To release attached bacteria, each well was treated with 1 mL of 1% (v/v) Triton X-100 in DPBS and incubated at 4^*o*^C for 30 min. The cell suspension including bacterial and Caco-2 cells were recovered from each well, centrifuged at 8,000 × *g* for 10 min at 4°C and washed two times with 1 mL of DPBS. Finally, the pellet was resuspended in PBS and serial dilutions were used to inoculate MRS agar media followed by incubation at 37^*o*^C for 48 h. Bacterial cell adhesion was expressed as percent bacterial CFU attached to Caco-2 cells, relative to the initial bacterial population added per well. The results correspond to the means of two independent experiments, three replicates each.

#### Auto-Aggregation

Auto-aggregation test was performed as described previously (Del Re et al., 2000) with modifications. Overnight cultures of *E. durans* OSY-EGY and *E. faecalis* symbioflor 1 were harvested and cell pellets were washed twice with sterile phosphate buffer saline (PBS, pH 7.0) and resuspended in the same buffer to obtain a cell suspension of approximately 10^8^ CFU/ml. Portions (4 ml each) of cell suspensions were incubated at 37°C for 24 h. One-milliliter aliquots of the supernatants at time 0 and 24 h of incubation without agitation were transferred to a spectrophotometer cuvette and absorbance (OD_600_) at these two points (A_0_ and A_24__*h*_, respectively) was measured. The percentage of auto-aggregation was then calculated as follows:

$$\text{Auto}\_\text{aggregation}\left(\%\right)=\left[1-\frac{{\text{A}}_{24\,\text{h}}\,}{{\text{A}}_{0}}\right]\times 100$$

The assay was performed in triplicate.

#### Hydrophobicity

Hydrophobicity test was performed as initially described (Rosenberg et al., 1980) with some modifications. Overnight cultures of *E. durans* OSY-EGY and *E. faecalis* symbioflor 1 were harvested and cell pellets were washed twice with sterile phosphate buffer saline (PBS, pH 7.0) and resuspended in the same buffer to obtain a cell suspension of approximately 10^8^ CFU/ml. One-milliliter of toluene (99.8%; Sigma-Aldrich) was added to 3 ml of each cell suspension, vortexed for 90 s and incubated at 37°C for 1h for phase separation. After incubation, OD_600__*nm*_ of aqueous phase was measured and hydrophobicity was calculated as follows:

$$\text{Hydrophobicity}\left(\%\right)=\left[1-\frac{\text{A}}{{\text{A}}_{0}}\right]\times 100$$

where A_0_ and A are the measured absorbance values before and after mixing with toluene, respectively. The assay was performed in triplicate.

#### Antioxidant Effect

The radical scavenging ability of 2,2-diphenyl-1-picrylhydrazyl (DPPH) was used to evaluate the antioxidant effect (Kedare and Singh, 2011). The assay was performed on *E. durans* OSY-EGY as described previously (Brand-Williams et al., 1995) with some modifications. The DPPH was freshly prepared in methyl alcohol at a final concentration of 60 μM and transferred to a dark glass bottle. Aliquots (100 μl) of filter-sterilized cell-free supernatant of *E. durans* OSY-EGY strain were mixed with 3.9 ml of DPPH radical solution. Methyl alcohol was used as a blank. Aliquots (100 μl) of water mixed with 3.9 ml of DPPH solution were prepared as control. The absorbance of the resulting solutions was measured at 515 nm at 5-min intervals for 65 min. The assay was performed in triplicate.

#### Cholesterol-Lowering Activity

Assimilation of cholesterol was determined as described previously (Choi and Chang, 2015) with slight modifications. Cholesterol (Sigma–Aldrich) was dissolved in 50% ethanol at final concentration of 5mg/ml, followed by filter sterilization using 0.45 μm micro-filter (Fisher Scientific). The soluble cholesterol was added to MRS broth supplemented with 0.3% bile salts, resulting in 0.1 g/l final concentration of cholesterol in MRS broth. Cholesterol-MRS broth was inoculated with *E. durans* OSY-EGY overnight culture at 10^8^ CFU/ml final cell density before anaerobic incubation at 37°C for 24h. After incubation, aliquots of *E. durans* OSY-EGY culture was centrifuged at 16,000 × *g* at 4°C for 5min before the supernatant was transferred for measuring residual cholesterol concentration. Cholesterol-MRS broth without *E. durans* cells was used as untreated control. Residual cholesterol concentration was determined using *o*-phthalaldehyde-based method (Rudel and Morris, 1973). Briefly, 1 ml of the supernatant was added to 2 ml of KOH (33%, w/v) and 3mL of 95% ethanol followed by shaking for 1 min and heating at 60°C for 10 min. After cooling in cold water, 5 ml hexane were added, followed by vortexing for 1 min, and adding 1 ml distilled water. The tube was held at ∼22°C for 10 min to allow phase separation followed by transferring 3 ml of the hexane layer into a clean glass tube for evaporation using speed vacuum dryer (Savant AES 2010 Rotary Evaporation System; Savant Inc., Holbrook, NY). Four milliliters of *O*-phthalaldehyde solution (0.5 mg of *O*-phthalaldehyde/ml of acetic acid; Sigma–Aldrich) were added to the residual cholesterol, and the mixture was vortexed and held at ∼22°C for 10 min. Subsequently, 2 ml concentrated sulfuric acid were added before incubation for 10 min at 25°C. The absorbance was then measured at 550 nm using a spectrophotometer (Spectronic 20 Genesys; Spectronic Instruments, Inc., Rochester, NY). Cholesterol removal was calculated from the equation:

$$\mathrm{Cholesterol}\mathrm{removal}\left(\%\right)=\left[1-\frac{{\text{A}}_{\text{s}}\,}{{\text{A}}_{\text{c}}}\right]\times 100$$

where A_*s*_ and A_*c*_ are the absorbance at 550 nm for *E. durans* OSY-EGY supernatant and control (un-inoculated Cholesterol-MRS broth), respectively.

### Antimicrobial Activity Assessment

The antimicrobial potential of *E. durans* OSY-EGY was assayed using the previously described spot-on-lawn method (He et al., 2007). Briefly, stock culture of *E. durans* OSY-EGY was streaked on MRS agar followed by incubation at 30°C for 48 h. Subsequently, a single colony was inoculated in MRS broth followed by incubation at 30°C for 48 h. The obtained fermentate was centrifuged at 7,710 × *g* for 15 min and the culture supernatant was filter-sterilized using 0.45-μm syringe filter (Millex HV Durapore PVDF; Merk Millipore, Ltd., Cork, IRL). The resulting cell-free supernatant (CFS) was assayed for antimicrobial efficacy against selected bacterial strains (Table 3) as follows. Aliquots (10 μl) of overnight cultures of tested bacteria were transferred into 10 ml sterile molten TSB soft agar (0.75% agar). The inoculated soft agar was overlayed onto basal TSA agar plates. After the soft agar solidified, 10 μl of the CFS were spotted on the indicator lawn. After overnight incubation at 37°C, the clear inhibitory areas, where the CFS was spotted, were observed.

**TABLE 3:** Antimicrobial activity of *Enterococcus durans* OSY-EGY against selected bacteria.

**Tested bacteria**	**Activity**
**Gram-positive**	
°*Bacillus cereus* ATCC 14579	+^*a*^
°*Enterococcus faecalis* ATCC 29212	+
°*Listeria monocytogenes* Scott A	+
°*Staphylococcus aureus* ATCC 29213	+
**Gram-negative**	
°*Escherichia coli* K12	–

*^*a*^Antimicrobial activity was observed as visible inhibition of growth during bioassay, as described in the materials and methods section.*

### Statistical Analysis

Statistical analysis was performed using R (see text footnote 5). Data were expressed as mean ± standard deviation (SD). Statistical significance was determined using one-way analysis of variance (ANOVA) for comparisons among treatments. Control-treatment comparisons were performed by Student’s t-test and significance was considered at *p* < 0.05.

## Results

A new lactic acid bacterium strain, *E. durans* OSY-EGY, was isolated from an artisanal cheese and found to have a promising antimicrobial activity that could be beneficial in food applications (Hussein et al., 2019). Before proposing the use of the strain in further industrial applications, research is needed to assess its safety, antimicrobial, and probiotic traits. This study, therefore, was initiated to thoroughly assess these traits using genomic and phenotypic analyses.

### Safety and Probiotic-Related Traits in *E. durans* OSY-EGY Genome

The draft genome of *E. durans* OSY-EGY was mined for genetic determinants associated with safety and probiotic traits. The strain was found lacking genes encoding hyaluronidase (*hyl*), cytolysin (*cylA*), gelatinase (*gelE*) and genes involved in biogenic amines biosynthesis except tyramine decarboxylase (*tdc*) for tyramine production. For immunity against mobile genetic elements, *E. durans* OSY-EGY genome was found to contain one confirmed and four potential CRISPR arrays. The genome, however, was found to contain genes involved in biosynthesis of the essential amino acids tryptophan, phenylalanine, methionine, valine, leucine, isoleucine, histidine, arginine and lysine (Table 4) and the vitamins pantothenate, thiamin, riboflavin and folate (Table 5).

**TABLE 4:** Essential amino acid and associated biosynthetic proteins detected in *Enterococcus durans* OSY-EGY genome.

**Amino acid**	**Biosynthesis Protein**
Tryptophan	Tryptophan synthase alpha chain Tryptophan synthase beta chain Indole-3-glycerol phosphate synthase Phosphoribosylanthranilate isomerase Anthranilate phosphoribosyltransferase Chorismate synthase 3-phosphoshikimate 1-carboxyvinyltransferase Shikimate kinase Shikimate dehydrogenase 3-dehydroquinate dehydratase I 3-deoxy-7-phosphoheptulonate synthase
Phenylalanine	Prephenate dehydratase Aspartate aminotransferase
Methionine	L-methionine (R)-*S*-oxide reductase Homocysteine *S*-methyltransferase Cysteine-*S*-conjugate beta-lyase
Valine	Branched-chain amino acid aminotransferase Valine-pyruvate aminotransferase
Leucine	Aminotransferase
Isoleucine	Aminotransferase
Histidine	Cytosolic non-specific dipeptidase
Arginine	Arginine deiminase Argininosuccinate lyase Argininosuccinate synthase ornithine carbamoyltransferase Carbamate kinase Glutaminase Glutamine synthetase Glutamate dehydrogenase (NADP +) Alanine transaminase
Lysine	Aspartate kinase Diaminopimelate decarboxylase Diaminopimelate epimerase Succinyl-diaminopimelate desuccinylase *N*-acetyldiaminopimelate deacetylase 2,3,4,5-tetrahydropyridine-2,6-dicarboxylate *N*-succinyltransferase 4-hydroxy-tetrahydrodipicolinate synthase Aspartate-semialdehyde dehydrogenase Aminotransferase
Cysteine	Cysteine synthetase Serine acetyl transferase

**TABLE 5:** Vitamins biosynthetic proteins detected in *Enterococcus durans* OSY-EGY genome.

**Vitamins**	**Biosynthesis protein**
Thiamine	Thiamine phosphate phosphatase
Riboflavin	5-amino-6-(5-phospho-D-ribitylamino) uracil phosphatase
Folate	Dihydrofolate reductase Folylpolyglutamate synthase Dihydroneopterin aldolase
Pantothenate	Type I pantothenate kinase Pantetheine-phosphate adenylyltransferase Phosphopantothenoyl cysteine decarboxylase Phosphopantothenate—cysteine ligase

### Comparative Genomic Analysis

**FIGURE 1:**
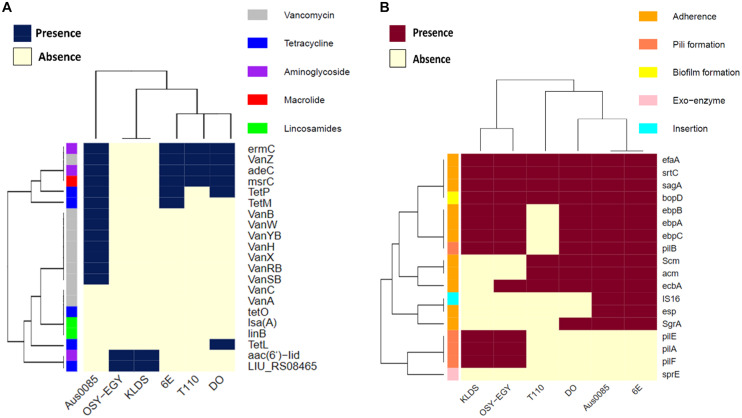
Heatmap showing the clustering of the compared *Enterococcus* spp. genomes based on the presence of **(A)** antibiotic resistance genes, and **(B)** virulence genes.

Genomic comparison was performed to reveal the differences between this strain or probiotic or pathogenic enterococci. When screened for the presence of antibiotic resistance genes (Figure 1A), *E. durans* OSY-EGY and *E. durans* KLDS6.0930 genomes were found to contain *aac(6’)-Iid* and LIU_RS08465 genes, which are associated with aminoglycosides and tetracycline resistance, respectively. The genome of the commercial probiotic T110 possessed genes involved in vancomycin, aminoglycosides and macrolides resistance. Genomes of the potentially pathogenic strains (DO, Aus0085, or 6E6) harbored genes conferring resistance to vancomycin, tetracycline, aminoglycosides, and macrolides. When screening for the presence of virulence genes (Figure 1B), *E. durans* OSY-EGY genome was void of the virulence factors *IS16*, *acm*, *Scm*, *SgrA* and *esp*. However, genes associated with adhesion (*ebpA*, *ebpB*, *ebpC*, *srtC*, *EfaA* and *sagA*), pilli formation (*pilA*, *pilB*, *pilE* and *pilF*) and biofilm formation (*bopD*) existed in all enterococcal genomes.

**FIGURE 2:**
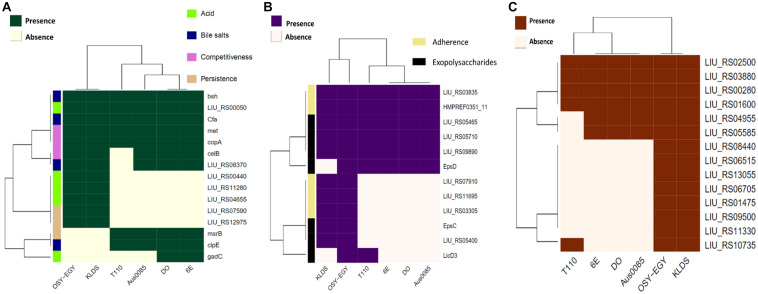
Heatmap showing the clustering of the compared *Enterococcus* spp. genomes based on the presence of **(A)** genes associated with survival in the GIT, **(B)** genes associated with adherence to the GIT, and **(C)** genes associated with antioxidant activity.

**FIGURE 3:**
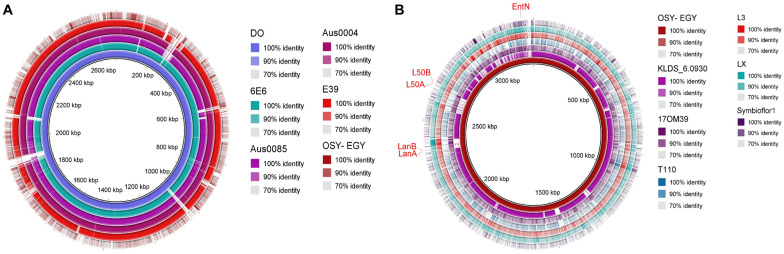
Blast Ring Image Generator (BRIG) diagram comparing *Enterococcus durans* OSY-EGY genome with genomes of **(A)** potentially pathogenic *Enterococcus* spp. using complete genome of *E. faecium* DO as a reference **(B)** probiotic *Enterococcus* spp. using draft genome of *E. durans* OSY-EGY as a reference.

The ability of probiotics to exert health benefits depends on their survival and persistence in the GIT. Genes encoding these traits such as acid and bile tolerance, competitiveness, persistence, and adherence were mined in the compared genomes. All studied genomes encoded acid tolerance, bile tolerance, competitiveness, and persistence genes (Figure 2A). Genes encoding cell adhesion-associated proteins existed in all genomes except those encoding S-layer protein and collagen binding proteins, which were found only in *E. durans* OSY-EGY and probiotic genomes. The strains OSY-EGY, KLDS6.0930, and T110 contained several genes involved in the biosynthesis of exopolysaccharides. The shortage of these genes in the potentially pathogenic strains may indicate the competitive advantage of the probiotic strains to colonize the GIT (Figure 2B). The *E. durans* OSY-EGY and KLDS6.0930 were found to share 14 genes associated with antioxidant activities, suggesting their potential ability to survive in the host environment. In comparison, only six genes associated with antioxidant activity were found in the potentially pathogenic genomes (Figure 2C). Detailed information about all genes used for comparison is listed in the Supplementary Tables 1–5. Genome of *E. durans* OSY-EGY was aligned with those of potentially pathogenic enterococci and results are shown in Figure 3A. This genomic comparison revealed how different and distantly removed is *E. durans* OSY-EGY from the potentially pathogenic strains. When aligning genomes of *E. durans* OSY-EGY with probiotic enterococci, it was found that *durans* KLDS6.0930, a well characterized potential probiotic (Li et al., 2018b), has the highest similarity to *E. durans* OSY-EGY. Compared to other genomes, *E. durans* OSY-EGY has the advantage of carrying genes encoding multiple bacteriocins; these are two novel sequences of lantibiotics A and B, enterocin N, and the two previously known enterocins, L50A and L50B (Figure 3B).

### Detection of Virulence Genes and Screening for Plasmids

For confirming the *in silico* analysis of selected virulence factors and biogenic amines production, PCR was conducted on the genomic DNA extracted from OSY-EGY and Symbioflor 1, using primers specific for the corresponding genes (Table 2). The results showed that *GelE*, *cylA*, *asa1*, *esp*, *hdc*, *odc* were absent in *E. durans* OSY-EGY and Symbioflor 1 genomes. *Ace*, *agg* and *hyl* genes were found only in Symbioflor 1 genome. The genes *EfaA* and *tdc* were found in the genomes of both *E. durans* OSY-EGY and Symbioflor 1 (Table 6), which confirmed the genomic analysis findings.

**TABLE 6:** Detection of selected virulence and biogenic amines production genes in the genomes of *Enterococcus durans* OSY-EGY and *E. faecalis* Symbioflor 1.

**Gene^*a*^**	***E. durans* OSY-EGY**	***E. faecalis* Symbioflor 1**
*efaA*	D^*b*^	D
*ace*	ND^*c*^	D
*agg*	ND	D
*asa1*	ND	ND
*esp*	ND	ND
*gelE*	ND	ND
*cylA*	ND	ND
*hyl*	ND	D
*odc*	ND	ND
*tdc*	D	D
*hdc*	ND	ND

*^*a*^Gene description is found in Table 2. ^*b*^Detected. ^*c*^Not detected.*

Plasmids include considerable portion of genomic information associated with antibiotic resistance and virulence factors. Plasmid DNA was extracted from *E. durans* OSY-EGY, Symbioflor 1 and *E. coli* Pet22b strains followed by visualization using gel electrophoresis. Unlike *E. coli* Pet22b, *E. durans* OSY-EGY and Symbioflor 1 were devoid of plasmids (Data are not shown).

### Phenotypic Assessment of Safety-Related Traits

**FIGURE 4:**
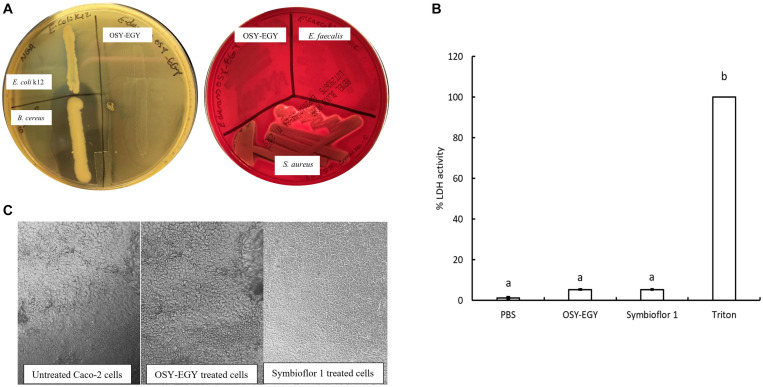
Safety traits of *Enterococcus durans* OSY-EGY. **(A)** Gelatinase activity (Left panel) of *E. durans* OSY-EGY compared to *Bacillus cereus* ATCC 14579 (positive control) and *Escherichia coli* k12 (negative control) on nutrient agar containing 3% gelatin and flooded with ammonium sulfate saturated solution. Clear zone indicates gelatin hydrolysis due to gelatinase production. Hemolytic activities (right panel) of *E. durans* OSY-EGY on blood agar compared to *S. aureus* ATCC 25923 as positive control and *E. faecalis* ATCC 29212 as negative control. Clear zone indicates red blood cells lysis due to hemolysin production. **(B)** Cytotoxic effect of *E. durans* OSY-EGY or *E. Faecalis* (Symbioflor 1) strains on Caco-2 cells after 24 h treatments. Values were expressed as means ± SD of two independent repeats. Treatments that were significantly different at *P* ≤ 0.05 were indicated by different letters. **(C)** Microscopy observation of Caco-2 cells after 3 h of treatment with *E. durans* OSY-EGY or *E. faecalis* Symbioflor 1 compared to untreated Caco-2 cells.

The minimum inhibitory concentrations (MICs) for nine antibiotics were determined against *E. durans* OSY-EGY (Table 7). Results show that the strain is susceptible to all tested antibiotics. The absence of cytolysin and gelatinase genes, revealed from the genomic analysis, was confirmed using the phenotypic assays. Unlike *B. cereus* ATCC 14579, *E. durans* OSY-EGY did not exhibit any gelatin hydrolysis (Figure 4A). When *E. durans* OSY-EGY was grown on blood agar, it did not exhibit any hemolytic effects, compared to *S. aureus* ATCC 25923 which produced strong β hemolysis (Figure 4A).

**TABLE 7:** The minimum inhibitory concentrations of selected antibiotics tested against *Eterococcus durans* OSY-EGY.

**Antibiotic**	**MIC**	Cut-off value*
Ampicillin	2	2
Clindamycin	2	4
Erythromycin	≤1	4
Kanamycin	512	1,024
Streptomycin	32	128
Tetracycline	4	4
Vancomycin	4	4
Gentamycin	32	32
Chloramphenicol	8	16

*The cut-off values are based on the guidelines of European Food Safety Authority (EFSA, 2012a) for the assessment of bacterial resistance to antibiotics.

After 24 h of treating Caco-2 cells with *E. durans* OSY-EGY or *E. faecalis* Symbioflor 1, the amount of LDH released was measured as an indicator of cytotoxic effect. The results showed that there was no significant difference (*p* > 0.05), in the LDH release (approximately 5%), between *E. durans* OSY-EGY, *E. faecalis* Symbioflor 1, or PBS-treated cells (Figure 4B). The microscopic observation did not show differences in the morphology of treated or untreated Caco-2 cells (Figure 4C).

### Phenotypic Assessment of Probiotic-Related Traits

**FIGURE 5:**
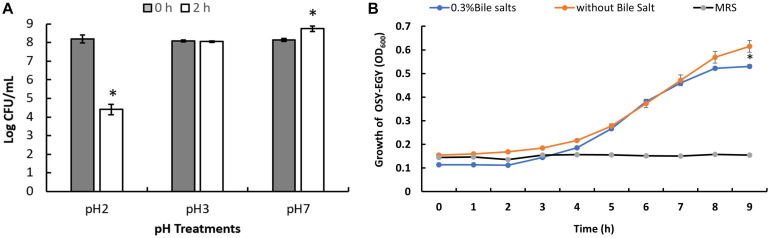
**(A)** Tolerance of *Enterococcus durans* OSY-EGY to acidic pH after 2 h of incubation at 37°C. Values were expressed as means ± SD of three repeats and values that were statistically different were indicated by asterisks (^∗^) if *p* < 0.05. **(B)** Tolerance of *E. durans* OSY-EGY to 0.3% bile salts after 9 h of incubation at 37°C under anaerobic conditions. Values were expressed as means ± SD of three repeats and values that were statistically different were indicated by asterisks (*) if *p* < 0.05.

When *E. durans* OSY-EGY was exposed to pH 3.0 for 2 h at 37°C, the strain population did not decrease significantly (*P* > 0.05) as shown in Figure 5A. However, there was a decrease in the population (*P* < 0.05) after holding *E. durans* OSY-EGY at pH 2.0 for 2 h. These results indicate that *E. durans* OSY-EGY can survive acidic conditions as low as pH 3.0. The strain OSY-EGY also grew uninhibited for 8 h in the presence of bile salt at a concentration of 0.3% (*p* > 0.05), as shown in Figure 5B.

**FIGURE 6:**
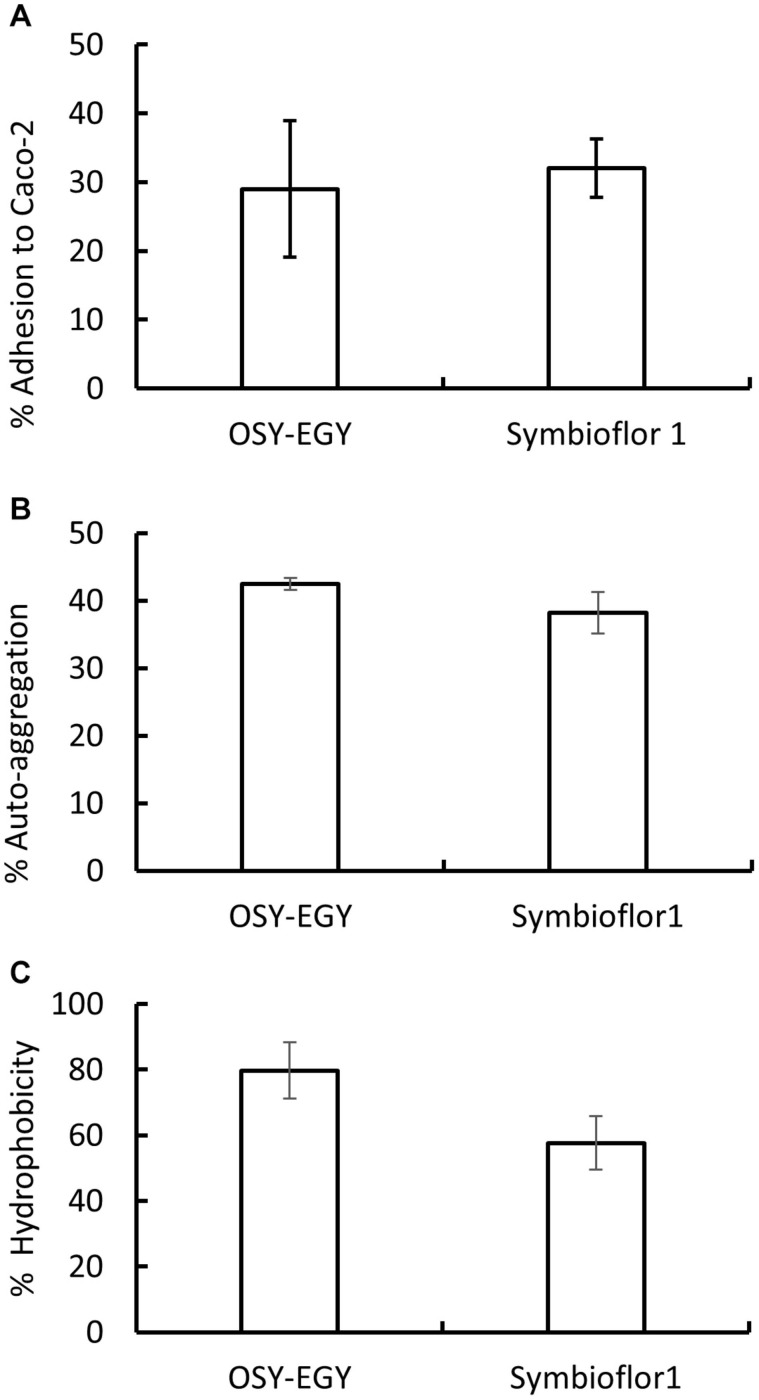
Adhesion capabilities of *Enterococcus durans* OSY-EGY and *E. faecalis* Symbioflor 1 strains. **(A)** Adherence to Caco-2 cells monolayer after 3 h of incubation. **(B)** Auto-aggregation capabilities after 24 h or incubation at 37°C, and **(C)** hydrophobicity measured as adhesion percentage to toluene after 1 h of incubation at 37°C. Values were expressed as means ± SD of three repeats.

OSY-EGY and Symbioflor 1 strains showed similar capabilities to adhere effectively to Caco-2 cells (Figure 6A). For additional adhesion characteristics, OSY-EGY and Symbioflor 1 were evaluated for auto-aggregation capabilities and hydrophobicity. The auto-aggregation capability was not significantly higher in OSY-EGY than in Symbioflor 1 strain (*p* > 0.05) (Figure 6B). Likewise, after 1 h of incubation with toluene, OSY-EGY strain showed non-significant increase in hydrophobicity than did Symbioflor 1 strain (Figure 6C).

**FIGURE 7:**
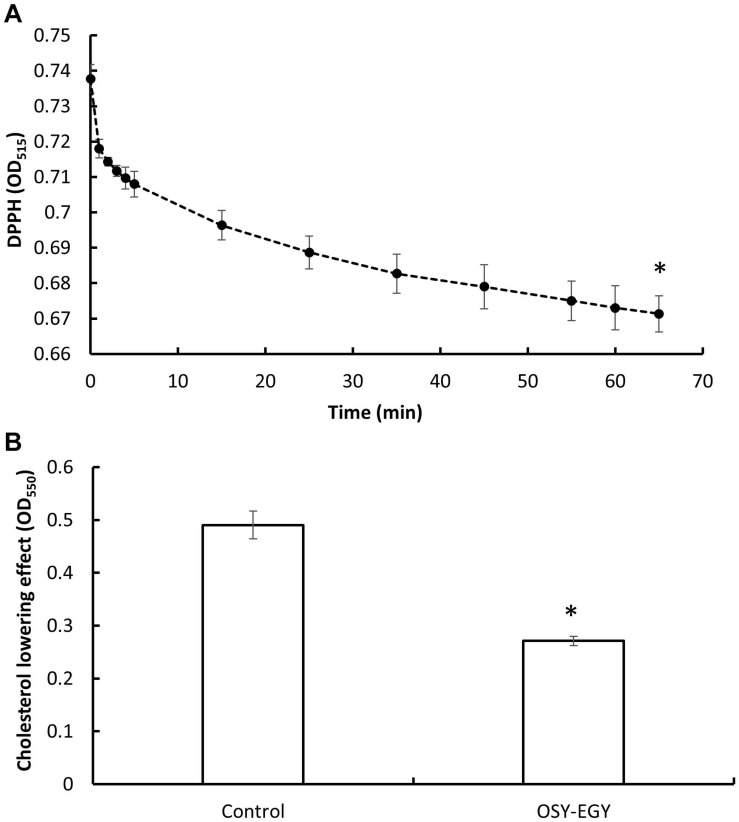
Antioxidant activity and cholesterol lowering effect of *Enterococcus durans* OSY-EGY. **(A)** Antioxidant activity measured by scavenging 2,2-diphenyl-1-picrylhydrazyl (DPPH) radicals. **(B)** Cholesterol lowering effect of *Enterococcus durans* OSY-EGY detected after incubation with cholesterol (0.1 g/L) and 0.3% (wt/vol) bile salt. Values were expressed as means ± SD of three repeats and values that were statistically different were indicated by asterisks (^∗^) if *P* < 0.05.

When evaluating the antioxidant activity, *E. durans* OSY-EGY showed DPPH radicals scavenging ability of 9% after 65 min of incubation, compared to the initial DPPH concentration (Figure 7A). The strain also had a significantly higher cholesterol lowering potential (*p* < 0.05), compared to the uninoculated control medium (Figure 7B). The CFS of *E. durans* OSY-EGY showed antimicrobial activity when tested against selected bacterial stains (Table 3); these include the pathogenic *B. cereus*, *L. monocytogenes*, and *S. aureus* and the spoilage *E. faecalis*. However, *E. durans* OSY-EGY was not effective against the Gram-negative *E. coli* K12.

## Discussion

*Enterococcus* spp. are known for their diversity, ubiquitous distribution in the environment, and presence in fermented foods (Ghattargi et al., 2018). There has been an extensive research interest in enterococci as probiotic candidates (Foulquié Moreno et al., 2006; Franz et al., 2011; Nami et al., 2014; Li et al., 2018b; Baccouri et al., 2019). To assess the safety and the industrial application feasibility of a strain, it is important to conduct genomic and phenotypic analyses on traits of interest. Emerging technologies such multi-locus sequence typing (Homan et al., 2002; Leavis et al., 2006; Ruiz-Garbajosa et al., 2006) and whole-genome sequence analyses (van Schaik et al., 2010; Qin et al., 2012; Beukers et al., 2017) have provided evidences that commensal enterococcal strains are different from the nosocomial infectious strains. Some of the genomic variability in *Enterococcus* spp. is due to the horizontal gene transfer leading to acquisition of mobile and foreign genetic elements such as bacteriophages and insertion sequence (IS) elements (Paulsen et al., 2003; Foulquié Moreno et al., 2006). Considering the potential hazards associated with gene transfer, new *Enterococcus* strains should be selected for industrial applications based on their genomes characterizations and absence of antibiotic resistance or virulence genes (Domann et al., 2007). A case-by-case safety evaluation assessment is critical to determine strain suitability for use as bio-protective culture or in probiotic preparations (Araújo and Ferreira, 2013; Hanchi et al., 2018).

In the current study, we found that *E. durans* OSY-EGY has a potent antimicrobial activity against Gram-positive, but not Gram-negative, bacteria. The strain was effective against *L. monocytogenes*, *S. aureus*, *E. faecalis*, and *B. cereus* and its genome was found to contain multiple bacteriocin biosynthetic gene clusters. Considering its promising antimicrobial activity, *E. durans* OSY-EGY has a potential to be used as bio-protective culture in some food fermentations. The presence of desirable probiotic characteristics is an additional advantage that may help in qualifying the strain for industrial applications. In terms of fitness and efficacy as a probiotic, antimicrobial activity is traditionally considered an important trait. Some researchers suggested that production of antimicrobials helps probiotic strains in competing against GIT microbes including pathogens (Gillor et al., 2009; Bosák et al., 2018).

In the current study, a genome analysis was conducted to assess the safety and probiotic traits in *E. durans* OSY-EGY, in comparison with known probiotic and potentially pathogenic *Enterococcus* strains (Ghattargi et al., 2018). BLAST Atlas revealed that *E. durans* OSY-EGY is closely related to the potential probiotic strain, KLDS6.0930, and distantly related to the potentially pathogenic group.

The antibiotic resistance is not uncommon in lactic acid bacteria (LAB), including strains proposed as probiotic candidates (Birri et al., 2012; Jeronymo-Ceneviva et al., 2014; Casarotti et al., 2017). In the GIT, there is a high potential for horizontal transfer of acquired antibiotic resistance genes among intestinal microbiota, including human pathogens. However, the horizontal transfer of intrinsic antibiotic resistance genes has a minimum potential (Devirgiliis et al., 2011; van Reenen and Dicks, 2011). EFSA requires the absence of acquired antibiotic resistance genes in bacterial strains, intended to be used in animal feed, as a critical part of its safety qualification (EFSA, 2012a). *In silico* analysis showed that *E. durans* OSY-EGY genome encodes genes that could be associated only with tetracycline (LIU_RS08465) and aminoglycoside (*aac(6′)-Iid*) resistance. In the current study, we screened, *in silico*, twenty *E. durans* genomes found in NCBI database for the presence of LIU_RS08465 and *aac(6’)-Iid* genes in an attempt to determine if these genes are associated with acquired antibiotic resistance. The results showed that these two genes were detected in all analyzed genomes (Supplementary Table 6) which indicates these antibiotic resistance genes are conserved in the genomes of *E. durans* and are associated with intrinsic resistances, with a low risk for lateral transfer (EFSA, 2012a). Additionally, we investigated the stability of *E. durans* OSY-EGY genome by analyzing for the presence of plasmids and CRISPER systems. *E. durans* OSY-EGY was found to be devoid of plasmids and its genome carries one confirmed and three potential CRISPR arrays which confers a sequence-based immunity and genome stability against phage modification and horizontal genes transfer (Marraffini and Sontheimer, 2008). Importantly, the antibiotic susceptibility test revealed that *E. durans* OSY-EGY is susceptible to clinically relevant antibiotics including ampicillin, vancomycin, tetracycline, and aminoglycosides. Although the commercial probiotic, *E. faecalis* Symbioflor 1, was used for more than 50 years without reporting of any adverse effect (Domann et al., 2007), it was previously reported to be gentamycin and erythromycin resistant (Baccouri et al., 2019).

According to EFSA guidelines, when the strain is ampicillin sensitive and the virulence genes IS*16*, *esp* and *hyl* are absent, the strain is considered safe to be used as feed additive (EFSA, 2012b); *E. durans* OSY-EGY genome lacks these markers. Absence of selected virulence genes in *E. durans* OSY-EGY genome was also verified using PCR. However, the commercial probiotic strain Symbioflor 1 contains the virulence genes, *ace* and *hyl*, in its genome. These findings support that *E. durans* OSY-EGY does not carry any potential pathogenicity. On the other hand, genes associated with adhesion, pilli formation and biofilm formation existed in all enterococcal genomes used for comparison (Figure 1B). The term virulence factor not only refers to elements that confer bacterial pathogenicity and development of infection but also to elements associated with cell adhesion and protection against host defense (Li et al., 2018b). However, factors involved in host cells adhesion and colonization of the intestine are needed in probiotics (Pillar and Gilmore, 2004; Wassenaar et al., 2015; Li et al., 2018b). The European Union recommended that the presence of confirmed known virulence genes in *Enterococcus* or other LAB strains should exclude these strains from use.

Before selecting new beneficial *Enterococcus* strain, its hemolytic activity and cytotoxic effect should be evaluated (Salminen et al., 1998). According to current study, *E. durans* OSY-EGY does not produce gelatinase or hemolysin. LDH assays are widely used for assessing the cytotoxic effect of proposed probiotic strains (Sanders et al., 2010; Hanchi et al., 2018). Using Caco-2 cells, coupled with LDH assay, to assess the cytotoxic effect of *E. durans* OSY-EGY and *E. faecalis* Symbioflor 1 showed that both strains had no cytotoxicity to this intestinal cell line. Members of LAB can synthesize biogenic amines from their precursor amino acids by amino acid decarboxylases (Benkerroum, 2016). Biogenic amines, including histamine, tyramine, ornithine, phenylethylamine and cadaverine, may cause intoxication if consumed in amounts that exceed critical thresholds (EFSA, 2011). When the genomes of *E. durans* OSY-EGY and *E. faecalis* Symbioflor 1 were screened for the presence of genes associated with biogenic amines production, both strains were void of all investigated genes except that encoding tyrosine decarboxylase, which is involved in tyramine production. Compared to *E. durans* KLDS6.0930, as the closest strain to OSY-EGY, KLDS6.0930 genome was found to contain the genes for tyramine production. However, KLDS6.0930 strain produced tyramine in amount less than the critical threshold level (Li et al., 2018b). Additionally, presence of the gene does not necessarily mean the protein is expressed; hence, further phenotypic validation of tyramine production needs to be conducted in a further study.

According to the World Health Organization (WHO), probiotics are ‘live microorganisms which when administered in adequate amounts confer a health benefit on the host’ (Food and Agriculture Organization [FAO], and World Health Organization [WHO], 2001). In order to confer such benefits, the probiotic strain should have the capacity to persist and survive under GIT conditions. The strain tolerance to acidic environment and bile salts are two criteria for a candidate probiotic strain to survive the GIT (Nami et al., 2019). In our study, we performed genomic and phenotypic analyses to confirm such traits in *E. durans* OSY-EGY. Genomically, *E. durans* OSY-EGY and the potential probiotic strain, KLDS6.0930, contained genes that encode proteins associated with counteracting acid stress such as tyrosine-tRNA ligase (Ladero et al., 2013), ATPase V, Na^+^/H^+^ antiporter, H^+^/K^+^ uptake transporter (Liu et al., 2016) and Tyrosyl-tRNA synthetase. The expression of the latter increases under low pH, suggesting its potential role in acid stress response (Linares et al., 2012). All *Enterococcus* genomes tested contain genes associated with bile tolerance, namely, genes encoding choloylglycine hydrolase and cyclopropane-fatty-acyl-phospholipid synthase. Transcription of the latter increased in lactic acid bacteria in response to bile (Budin-Verneuil et al., 2007; Hamon et al., 2011). On the other hand, *E. durans* OSY-EGY showed acid tolerance when grown at pH 2 or 3 and did not encounter growth reduction in the presence of 0.3% bile salts. This augments the potential capability of the strain to survive similar harsh conditions found in the GIT.

Colonization in the GIT is a critical trait to be considered for a potential probiotic strain. All genomes studied in this work were found to encode for cell adhesion factors such as fibronectin-binding protein (Hymes and Klaenhammer, 2016), aggregation-promoting factor that could be contributing to gut milieu binding (Ladero et al., 2013), collagen-binding protein and proteins associated with extracellular polymeric substance production which enhance adhesion and persistence in the gut environment (Liu et al., 2016). Phenotypically, *E. durans* OSY-EGY showed adhesion capabilities to Caco-2 cells. Cell surface hydrophobicity is an additional characteristic associated with the adhesion capability of the probiotic strains. The higher ability of probiotics to adhere to epithelial cells is a result of higher cell surface hydrophobicity (Xu et al., 2009). In the current study, *E. durans* OSY-EGY and *E. Faecalis* (Symbioflor 1) showed worthy hydrophobicity characteristics (Figure 6C). The auto-aggregation is defined as same-species bacterial accumulation and the trait is important in aiding cellular adhesion (Collado et al., 2008; Campana et al., 2017). In current study, *E. durans* OSY-EGY and *E. Faecalis* Symbioflor 1 showed promising auto-aggregation abilities (Figure 6B). Altogether, these results suggest that *E. durans* OSY-EGY most probably would survive GIT harsh conditions.

Probiotic desirable traits may include production of antioxidants, vitamins, and essential amino acids, and expression of enteric pathogens antagonism. *E. durans* OSY-EGY and other six genomes were screened for genes known to be associated with antioxidant effect. *E. durans* OSY-EGY and KLDS6.0930 shared 14 genes coding for antioxidant traits and only 5 genes were found in the potentially pathogenic strains. Genomes of pathogenic strains may include genes associated with GIT survival and even some beneficial characteristics. However, the presence of known virulence factors and antibiotic resistance is what makes these strains pathogenic to the host (Ghattargi et al., 2018). Consistent with its antioxidant characteristic, *E. durans* OSY-EGY showed DPPH radical scavenging effect (9%). Compared to *E. durans* OSY-EGY, *E. durans* F3 showed better DPPH scavenging activity (more than 40%) (Alshammari et al., 2019). This could be attributed to strain-specific differences in their antioxidant activities or the types of the antioxidants produced (Wang et al., 2017). *E. durans* OSY-EGY genome was found to have genes involved in biosynthetic pathways of essential amino acids and vitamins. The synthesis pathways for vitamins and amino acid were reported previously to be absent in the genomes of the potentially pathogenic strains DO, Aus0085, and 6E6 of *E. faecium* (Ghattargi et al., 2018). Other health-promoting benefit such as cholesterol lowering effect was observed in *E. durans* OSY-EGY (Figure 7B). Removal of cholesterol and biosynthesis of vitamins and essential amino acids further support the potential applications of *E. durans* OSY-EGY in promoting health and possibly in developing nutrient-rich foods. The promising antimicrobial activity of *E. durans* OSY-EGY against Gram-positive pathogenic and spoilage bacteria, indicates its usefulness for several industrial applications.

## Conclusion

Considering the antimicrobial effect of *E. durans* OSY-EGY, it was important to investigate its safety and to ensure its suitability for use in the industry as bio-protective or probiotic culture. The *in silico* comparative genomic analysis determined how distant is *E. durans* OSY-EGY from potentially pathogenic strains, as evident by the absence of antibiotic resistance and virulence genes. The analysis revealed genes associated with desirable probiotic traits such as acid tolerance, bile tolerance, competitiveness, persistence, adherence and health promoting effect. The functionality of the predicted genomic features was validated using a battery of *in vitro* analyses. From the findings of the current study, it can be concluded that *E. durans* OSY-EGY is potentially safe and beneficial strain that possesses desirable probiotic traits, in addition to its promising antimicrobial activity. This support the notion that the strain is potentially suitable for use in various applications.

## Data Availability Statement

The datasets presented in this study can be found in online repositories. The names of the repository/repositories and accession number(s) can be found in the article/Supplementary Material.

## Author Contributions

WH analyzed the strain phenotypically and genetically, drafted the manuscript. AA helped with genomic and bioinformatic analyses and contributed to the phenotypic and antibiotic susceptibility testing, and writing/revising the manuscript. DR-M carried out the experiments on cell-line. IG-C helped with the tissue culture work and completed the plasmid isolation experiment. AY oversaw the project and reviewed and revised the manuscript. All authors read and proved the manuscript.

## Conflict of Interest

The authors declare that the research was conducted in the absence of any commercial or financial relationships that could be construed as a potential conflict of interest.

## Footnotes

1 http://www.mgc.ac.cn/VFs/main.htm

2 http://www.genome.jp/kegg/kaas/

3 https://www.genome.jp/kegg/mapper/

4 http://crispr.i2bc.paris-saclay.fr/Server/

5 http://www.R-project.org/
