# Supplementary material for: Assessment of Safety and Probiotic Traits of Enterococcus durans OSY-EGY, Isolated From Egyptian Artisanal Cheese, Using Comparative Genomics and Phenotypic Analyses
Source: Front Microbiol. 2020 Dec 11;11:608314. doi: 10.3389/fmicb.2020.608314 (PMC7759505; doi:10.3389/fmicb.2020.608314)
Supplement: Supplementary file 1 [file Data_Sheet_1.PDF]

Table S1. Genes responsible for antibiotic resistance, detected in genomes of various strains of *Enterococcus* spp.

Category	Gene	Reference Gene ID[a]	OSY-EG	KLDS 6.0930	T110	NRRL B-2354	DO	Aus0085	6E6	Gene description
Vancomycin resistance	*VanA*	10164669[1]	ND[b]	ND	ND	ND	ND	ND	ND	D-alanine D-lactate ligase
	VanW	1201168[2]	ND	ND	ND	ND	ND	100/95[c]	ND	Vancomycin B type resistance protein
	VanB	1201166[2]	ND	ND	ND	ND	ND	100/96	ND	D-alanine D-lactate ligase
	VanYB	1201169[2]	ND	ND	ND	ND	ND	100/94	ND	D-alanyl-D-alanine carboxypeptidase
	VanH	1201167[2]	ND	ND	ND	ND	ND	99/95	ND	D-specific alpha keto acid dehydrogenase
	VanX	1201165[2]	ND	ND	ND	ND	ND	100/96	ND	D-alanyl-D-alanine dipeptidase
	VanRB	1201171[2]	ND	ND	ND	ND	ND	100/95	ND	DNA-binding response regulator
	VanSB	1201170[2]	ND	ND	ND	ND	ND	99/96	ND	Sensor histidine kinase
	VanZ	12998681[3]	ND	ND	100/100	100/100	100/100	100/100	100/100	Teicoplanin resistance protein vanZ
	VanC	AF162694.1[4]	ND	ND	ND	ND	ND	ND	ND	Vancomycin resistance gene cluster
Tetracycline resistance	LIU_R S08465	31916722[5]	100/99	100/100	ND	ND	ND	ND	ND	Tetracycline resistance MFS efflux pump

	TetP	12998658[3]	ND	ND	ND	ND	100/100	100/95	100/95	Tetracycline resistance protein
	TetL	12998659[3]	ND	ND	ND	ND	100/100	ND	ND	Tetracycline cation symporter
	TetM	29476824[6]	ND	ND	ND	ND	ND	100/98	100/98	Tetracycline resistance ribosomal protection protein
	tetO	29676526[6]	ND	ND	ND	ND	ND	ND	ND	Tetracycline resistance protein
Aminoglycoside resistance	*ermC*	12998871[3]	ND	ND	100/94	100/99	100/100	100/100	100/100	Erythromycin resistance leader peptide ErmC
	aac(6')-Iid	31917481[5]	100/100	100/100	ND	ND	ND	ND	ND	Aminoglycoside acetyltransferase
	adeC	13001171[3]	ND	ND	99/93	100/100	100/100	100/100	100/100	Adenine deaminase
Macrolide	*msrC*	AY004350.1[7]	ND	ND	99/97	100/99	100/99	100/99	100/99	Acquired resistance like protein
Lincosamid-es	*lsa(A)*	AY225127.1[8]	ND	ND	ND	ND	ND	ND	ND	Putative efflux of lincosamides and streptogramins
	linB	AJ238249.1[9]	ND	ND	ND	ND	ND	ND	ND	Nucleotide transferase

[a]Genes were retrieved from *E. faecalis* S117[1], *E. faecalis* V583[2], *E. faecium* DO[3], *E. gallinarum* BM4174[4], *E. durans* KLDS6.0930[5], *E. cecorum* SA3[6], *E. faecium* TX2465[7], *E. faecalis* ATCC29212[8], *E. faecium* HM1025[9]. Sequence similarity analysis was performed using BLASTN.

[b]Gene was not detected

[c]Percent sequence coverage/identity

Table S2. Putative virulence genes, detected in genomes of various strains of *Enterococcus* spp.

Category	Gene	Reference Gene /Protein ID[a]	OSY-EG	KLDS 6.0930	T110	NRRL B-2354	DO	Aus0085	6E6	Gene description
Adherence	*acm*	YP_006376887[1**]	ND[b]	ND	100/98[c]	100/99	100/100	100/100	100/100	Collagen binding
	ebpA	YP_006376000[1**]	100/83	99/57	42/22	93/84	100/95	100/95	100/95	PilB-type pili subunit
	ebpB	YP_006375999[1**]	100/95	100/50	98/36	100/98	100/97	100/97	100/97	PilB-type pili subunit
	ebpC	YP_006375998[1**]	100/97	100/72	100/30	100/99	100/98	100/98	100/98	PilB-type pili subunit
	srtC	YP_006375997[1**]	84/80	83/73	93/87	93/93	93/94	93/94	93/94	Putative pilus dedicated sortase
	ecbA	YP_006376434[1**]	100/92	ND	ND	ND	100/100	100/100	100/95	Cell surface protein
	efaA	YP_006375106[1**]	99/74	97/75	100/95	100/100	100/100	100/100	100/100	Endocarditis associated adhesion protein
	Scm	YP_006377279[1**]	ND	ND	66/100	66/100	66/100	66/100	66/100	Second collagen adhesion protein
	SgrA	13000214[1*]	ND	ND	ND	ND	100/100	100/100	100/100	Cell surface protein
	sagA	13001297[1*]	78/85	78/85	100/93	100/100	100/100	100/99	100/100	Adhesion protein
	esp	AFC64830.1[2**]	ND	ND	ND	ND	ND	100/79	100/80	Enterococcal surface protein
Pili formation	*pilA*	ACI49671[3**]	100/96	93/27	72/27	93/26	95/26	95/26	95/26	PilA type pili subunit
	pilB	ACI49665[3**]	100/97	100/72	100/30	100/98	100/98	100/98	100/98	PilB type pili subunit
	pilE	ACI49673[3**]	100/81	70/30	50/28	58/24	60/29	60/29	60/29	Secreted surface protein
	pilF	ACI49668[3**]	100/97	10/48	6/41	6/41	6/41	6/41	6/41	Minor pilin subunit

Biofilm formation	*bopD*	12999074[1*]	100/100	100/100	98/95	100/99	100/100	100/99	100/100	Sugar binding transcriptional regulator
Exo-enzyme	*sprE*	ACO94087[4**]	ND	ND	ND	ND	ND	ND	ND	Serine protease
Insertion sequence	*IS16*	4594996[5*]	ND	ND	ND	ND	100/100	100/100	100/100	Mobile insertion sequence

[a]Genes or proteins were retrieved from *E. faecium* DO[1], *E. faecium* AUS0004[2], *E. faecium* E1165[3], *E. faecium* QSE32[4], and *E. faecium* U37[5]. Sequence similarity analysis was performed using BLASTN* or TBLASTN**.

[b]Gene was not detected

[c]Percent sequence coverage/identity

Table S3. Genes associated with survival in the gastro-intestinal tract and detected in genomes of various strains of *Enterococcus* spp.

Category	Gene	Reference Gene /Protein ID[a]	OSY-EG	KLDS 6.0930	T110	NRRL B-2354	DO	Aus0085	6E6	Gene description
Acid resistance	LIU_RS00050	31915068[*1]	100/100[b]	100/100	100/90	100/90	100/90	100/90	100/90	Tyrosine-tRNA ligase
	LIU_RS11280	31917264[*1]	100/99	100/100	ND[c]	ND	ND	ND	ND	ATPase V
	LIU_RS00440	31915144[*1]	100/100	100/100	ND	ND	ND	ND	ND	H+-K+ uptake transporter
	LIU_RS04655	31915971[*1]	100/100	100/100	ND	ND	ND	ND	ND	Na+/H+ antiporters
	gadC	12998919[*2]	ND	ND	ND	ND	100/100	ND	100/99	Organocation transporter
Bile resistance	*bsh*	AHH25138[**1]	99/92	99/64	99/92	99/91	99/92	99/92	99/92	Choloylglycine hydrolase
	Cfa	13000123[*2]	100/85	100/85	100/97	100/100	100/100	100/99	100/99	Cyclopropane-fatty-acyl-phospholipid synthase
	LIU_RS08370	31916703[*1]	100/99	100/100	ND	100/85	100/85	100/85	100/85	Cyclopropane-fatty-acyl-phospholipid synthase
	clpE	1199606[*3]	ND	ND	94/78	94/78	94/78	94/78	94/78	ATP dependent Clp protease
Competitiveness	*met*	ASV94521[**4]	100/99	100/99	100/94	100/94	100/94	100/94	100/94	Methionine-TRNA ligase
	celB	YP_006375666[**2]	98/94	98/94	99/37	100/99	100/100	100/99	100/99	PTS system cellobiose transporter
	copA	AGS75886[**5]	99/79	99/79	100/96	100/98	100/98	100/98	100/98	Copper –translocating ATPase
Persistence	*msrB*	12998799[*2]	ND	ND	100/93	100/100	100/100	100/100	100/100	Peptide-methionine (R) -S oxide reductase

LIU_RS07590	31916551[*1]	100/99	100/100	ND	ND	ND	ND	ND	NADH oxidase
LIU_RS12975	31917597[*1]	100/99	100/100	ND	ND	ND	ND	ND	NADH peroxidase

[a]Genes or proteins were retrieved from *E. durans* KLDS6.0930 [1], *E. faecium* DO[2], *E. faecalis* V583[3], *E. durans,* BDGP3[4] and *E. faecium* AUS0085[5], Sequence similarity was performed using BLASTN* or TBLASTN**.

[b]Percent sequence coverage/identity

[c]Gene was not detected

Table S4. Genes associated with adherence to gastro-intestinal tract and detected in genomes of various strains of *Enterococcus* spp.

Category	Gene	Reference Gene /Protein ID[a]	OSY-EG	KLDS 6.0930	T110	NRRL B-2354	DO	Aus0085	6E6	Gene description
Adherence	HMPREF0351_11907	13000603*1	97/96[b]	97/96	100/95	100/99	100/100	100/99	100/99	Fibronectin binding protein
	LIU_RS11695	31917346*2	100/99	100/100	ND[c]	ND	ND	ND	ND	S-layer protein
	LIU_RS07910	31916613*2	100/99	100/100	ND	ND	ND	ND	ND	Fibronectin binding protein
	LIU_RS03305	31915706*2	100/99	100/100	ND	ND	ND	ND	ND	Collagen adhesion protein
	LIU_RS03835	31915811*2	100/100	100/100	100/84	100/84	100/84	100/84	100/84	Aggregation-promoting factors
Exopolysaccharides										
	LIU_RS05465	31916131*2	99/99	100/100	100/80	99/80	99/99	99/99	99/99	Undecaprenyl-phosphate galactose phosphotransferase
	EpsC	31916983*2	100/100	100/100	ND	ND	ND	ND	ND	Tyrosine-protein kinase transmembrane modulator
	EpsD	AGE29659.1**3	100/88	96/53	100/92	100/94	100/89	100/90	100/90	Tyrosine-protein kinase
	LicD3	STD33019.1**4	100/78	97/58	100/99	100/100	ND	ND	ND	Lipopolysaccharide cholinephosphotransferase
	LIU_RS05400	31916118*2	100/99	100/100	ND	ND	ND	ND	ND	Family 2 Glycosyl transferase
	LIU_RS05710	31916180*2	100/100	100/100	96/79	97/79	97/79	97/79	97/79	Family 2 Glycosyl transferase
	LIU_RS09890	31916993*2	100/100	100/100	100/80	100/80	100/80	100/80	100/80	UDP-glucose4-epimerase

[a]Genes or proteins were retrieved from *E. durans* KLDS6.0930 [1], *E. faecium* DO[2], *E. faecalis* V583[3], *E. durans,* BDGP3[4] and *E. faecium* AUS0085[5], Sequence similarity was performed using BLASTN* or TBLASTN**.
[b]Percent sequence coverage/identity. [c]Gene was not detected

Table S5. Genes associated with antioxidant activity and detected in genomes of various strains of *Enterococcus* spp.

Category	Gene name	Reference Gene ID[a]	OSY-EGY	KLDS 6.0930	T110	NRRL B-2354	DO	Aus0085	6E6	Gene description
Antioxidant activity	LIU_RS05585	31916155	100/99[b]	100/100	ND[c]	100/84	100/84	100/84	100/84	Manganese superoxide dismutase
	LIU_RS03880	31915820	100/99	100/100	98/82	98/82	98/82	98/82	98/82	Manganese catalase
	LIU_RS06515	31916339	100/100	100/100	ND	ND	ND	ND	ND	Methionine sulfoxide reductase
	LIU_RS02500	31915552	100/100	100/100	99/87	99/87	99/87	99/87	99/87	Methionine adenosyltransferase
	LIU_RS00280	31915112	100/100	100/100	99/82	99/82	99/83	99/83	99/83	S-Adenosylmethionine ribosyltransferase
	LIU_RS08440	31916717	100/99	100/100	ND	ND	ND	ND	ND	Alkyl hydroperoxide reductase
	LIU_RS13055	31917613	100/100	100/100	ND	ND	ND	ND	ND	Glutathione peroxidase
	LIU_RS06705	31916377	100/99	100/100	ND	ND	ND	ND	ND	Glutathione reductase
	LIU_RS01475	31915348	100/100	100/100	ND	ND	ND	ND	ND	Glutathione-disulfide reductase
	LIU_RS09500	31916918	100/99	100/100	ND	ND	ND	ND	ND	Thioredoxin
	LIU_RS10735	31917158	100/99	100/100	99/82	ND	ND	ND	99/82	Thioredoxin-disulfide reductase
	LIU_RS11330	31917274	99/98	100/100	ND	ND	ND	ND	ND	Thiol reductase thioredoxin
	LIU_RS04955	31916030	100/100	100/100	ND	92/85	95/84	95/84	92/85	Redox-sensing transcriptional repressor

	LIU_RS01600	3195373	100/99	100/100	100/85	100/85	100/85	100/85	100/85	DNA starvation/stationary phase protection protein/DNA-binding ferritin-like protein

[a]Genes were retrieved from *E. durans* KLDS6.0930, and sequence similarity was performed using BLASTN.
[b]Percent sequence coverage/identity
[c]Gene was not detected

Table S6. Presence of genes associated with tetracycline and aminoglycosides resistance in all the sequenced genomes of *Enterococcus durans* retrieved from NCBI database

Strain	Query cover/ Percent identity	
	LIU_RS08465	*aac(6')-Iid*
OSY-EGY	100/99.9	100/100
IPLA 655	100/99.9	100/100
IQ23	100/99.8	100/100
18S	100/99.8	100/100
NCTC8130	100/99.8	100/100
NCTC8129	100/96.7	100/99.5
NBRC 100479	100/99.9	100/99.8
KLDS 6.0933	100/99.1	100/100
KLDS 6.0930	100/100	100/100
C11	100/100	100/100
ATCC 6056 V1	100/99.9	100/99.8
ATCC 6056 V2	100/99.9	100/99.8
AF11-32-H	100/96.3	100/99.5
FDAARGOS_396	100/99.9	100/99.8
am_0171	100/100	100/100
F0321E 104	100/96.3	100/99.5
BDGP3	100/96.3	100/99.5
CVMN59549F	100/95.7	100/97.5
4EA1	100/96.6	100/99.1
P16 CL A28	100/95.7	100/97.5
